# Association between Tobacco Consumption and Problematic Internet Use and the Practice of Physical Activity in Spanish Adolescents

**DOI:** 10.3390/ijerph18105464

**Published:** 2021-05-20

**Authors:** Víctor José Villanueva-Blasco, José Luis García-Soidán, Manuel Isorna Folgar, Víctor Arufe Giráldez

**Affiliations:** 1 Faculty of Health Sciences, Valencian International University, C/Pintor Sorolla, 21, 46002 Valencia, Spain; vjvillanueva@universidadviu.com; 2 Faculty of Education and Sport Sciences, Campus A Xunqueira s/n, University of Vigo, 36005 Pontevedra, Spain; jlsoidan@uvigo.es; 3 Faculty of Education and Social Work, Campus As Lagoas, University of Vigo, 32004 Ourense, Spain; 4 Facultad de Ciencias de la Educación, Universidad de A Coruña, 15008 A Coruña, Spain; v.arufe@udc.es

**Keywords:** adolescence, physical activity, tobacco, health, internet, sports federation

## Abstract

The practice of physical activity (PA) is a healthy habit that offers health benefits. In contrast, the lack thereof may be associated with an increase in diseases, even at an early age. The objective of this study was to analyze the association between unhealthy behaviors, such as tobacco consumption and problematic internet use, and the practice of PA in adolescents. Protective factors (physical activity and sport) and risk factors (leading a sedentary life, tobacco use, and problematic internet use) were evaluated. Other variables such as sex, the intensity of physical activity, and being a member of a sports federation were also evaluated. The sample consisted of a total of 1222 Spanish adolescents. Univariate descriptive analysis and multiple linear regression were used, and confirmatory factor analyses and structural models were also estimated. The results confirm a significant positive association between physical activity, intensity, and being a member of a sports federation, as well as between cigarette consumption and internet use. It is advisable to implement public policies that promote the practice of sports as a direct investment in health, preventing the consumption of tobacco and other habits that are harmful to the health of adolescents.

## 1. Introduction

The practice of physical activity (PA) and sports is a healthy habit that offers well-being and positive health benefits at a physical and psychological level [1]. In contrast, the lack of PA is associated with increased health risks in the adolescent population [2,3,4]. Although international organizations such as the Physical Activity Guidelines Advisory Committee [5] or the World Health Organization (WHO) [6,7] have established PA recommendations for children and adolescents to avoid a sedentary lifestyle (i.e., at least 60 min per day of moderate-vigorous physical activity; MVPA), and these have been adopted by many countries [8,9], they are often unknown or neglected by the beneficiaries. In the global report on physical activity in children and adolescents between 5 and 17 years old, conducted in 49 countries on 6 continents, it is reported that the degree of PA compliance had a “low/poor” D level (between 27–33%) [10].

The transitional stage between childhood and adolescence appears to be a critical time in relation to different health-related behaviors. This stage is associated with a progressive decrease in PA levels [11,12], as well as an increase in sedentary screen time [13,14]. It is also when the consumption of alcohol, tobacco, and illicit drugs begins [15,16]. According to the ESTUDES survey [17], 41.3% of students aged 14 to 19 have ever smoked tobacco, 35% in the last year and 26.7% in the 30 days prior to the survey. The prevalence of daily tobacco use is also 9.8%.

Although the impact of these unhealthy habits in the form of chronic diseases may not emerge until adulthood, health risk behaviors often begin to develop in childhood and adolescence [18]. In this sense, it is interesting to know whether these risk behaviors are related to each other, posing a common challenge from the comprehensive approach to health.

Some studies and reviews indicate that the practice of PA in adolescents is associated with lower tobacco consumption [19,20], while adolescents with sedentary lifestyles are more likely to be smokers [21,22,23]. However, other studies do not find a significant relationship [24] or indicate that smoking and PA are independent behaviors [25].

Regarding gender differences, some studies point to the association of greater PA and sports practice with lower substance use regardless of sex [21,26], while others associate it with higher substance use in boys but not in girls [22,27].

A recent study [28] has confirmed a growing consumption of substances such as tobacco among federated athletes and especially among the youngest, despite their serious adverse health effects. Although the consumption of legal drugs is less frequent in athletes, some researchers [29] indicate that it depends on the sport practiced and the level of competition, noting that there is a greater association with tobacco use in athletes practicing team sports.

In turn, the improper use of information and communication technologies (ICT) would also constitute a risk factor for the development of sedentary lifestyles [30,31,32,33,34,35]. However, Pearson et al., [36], through a meta-analysis in which 163 studies were included, found a small negative association between sedentary screen time and the practice of PA in children and adolescents. This finding suggests that one behavior does not displace the other and that they can coexist simultaneously. More specifically, problematic internet use (PIU) is more prevalent among adolescents who do not practice sport (19.2%) compared to those who practice sport or non-regulated physical activity (11.5%) and to a lesser extent among those who practice federated sport in a club (7.7%) [37].

Numerous studies indicate that PA levels [38,39], sedentary screen time, and tobacco consumption [40] adopted in the early stages tend to be maintained in youth and adulthood. Therefore, adolescence is a relevant stage in relation to the promotion of healthy habits, where being a member of a sports federation is understood as a factor that ensures an adequate level of PA under supervision.

Our objective was to examine the association between unhealthy behaviors, such as smoking and problematic internet use, and the practice of PA and its intensity in adolescents. Based on what is indicated by the literature, analyzing the role of sex was considered with the fact of being a member of a sports federation as a mediating variable. The research questions were: (a) Is a greater presence of unhealthy habits (smoking, PIU) related to a lower AF and intensity of this? (b) Would there be gender differences in these hypothetical associations? (c) Does being a member of a federated sport have any moderating effect between these unhealthy habits and FA and its intensity?

## 2. Materials and Methods

### 2.1. Study Design and Participants

The study is cross-sectional with a sample of students from different schools and different levels of education belonging to eight public schools in the cities of Pontevedra, Valencia, Madrid, A Coruña, Las Palmas de Gran Canaria, and Teruel (Spain). Random sampling was used for the selection of the schools, confirming their participation in the study with the Directorate of the educational center. Subsequently, convenience sampling was used at the group-classroom level.

The sample size was estimated to perform a path analysis, considering the number of items in the instruments. The sample was initially composed of 1421 participants, but 14% were eliminated due to incoherent response patterns, missing values, or because they were outside the age range under study (between 12 and 18 years old). The final sample consisted of 1222 students, 589 boys (47.8%) and 643 girls (52.2%), with a mean age of 14.98 years (SD = 1.68). A total of 13.7% were in the 1st year of junior high school, known in Spain as compulsory secondary education (ESO); 24.4% in the 2nd year of E.S.O; 14.1% in the 3rd year of ESO; 21.2% in the 4th year of ESO; 23.2% in high school (Bachillerato); and 3.4% in vocational training (ciclos formativos).

The inclusion criteria were to be enrolled in a school in Spain and to be between 12 and 18 years of age. Ethnicity, socioeconomic level, and religion were not exclusion criteria.

### 2.2. Instruments

Different instruments were used for data collection, as described below.

#### 2.2.1. Sociodemographic Questionnaire

This ad hoc instrument collected data on the school, school ownership, the reference sample unit, the assigned confidential alphanumeric code, gender (Boy = 1; Girl = 2) and age of the participants.

#### 2.2.2. Survey on Drug Use in Secondary Education in Spain (ESTUDES)

The survey on Drug Use in Secondary Education in Spain (ESTUDES) [41] was used The question “How many cigarettes do you smoke per day, on average?” was used to establish a gradation of tobacco use. It was measured according to the following ranges: (0) None; (1) Fewer than 10 cigarettes/day; (2) Between 11–20 cigarettes/day; (3) Between 21–30 cigarettes/day; and (4) 31 or more cigarettes/day.

#### 2.2.3. Problematic Internet Use in Adolescents Scale (PIUS-a) 

The PIUS-a [42] is composed of 11 items (e.g., “On occasion I have come to neglect some tasks or to perform less (in exams, sports, etc.) by connecting to the internet”) that are answered on a Likert-type scale of five alternatives ranging from 0 (do not agree at all) to 4 (strongly agree). This instrument allows adolescents to be scaled on a continuum of risk of problematic internet use. The total score ranges from 0 to 44. The cut-off point established for the detection of problematic use is set at 16 points, so adolescents with an equal or higher score are classified as problematic users. It has satisfactory psychometric properties in terms of reliability of the scores (α = 0.82), evidence of its internal structure (tested via a confirmatory factorial analysis), sensitivity (81%), and specificity (82.6%). Cronbach’s alpha index of 0.83 being found in this sample.

#### 2.2.4. Physical Activity Level, “Finnish Physical-Sport Activity Index” 

The five items on the scale Finnish Physical-Sport Activity Index [43,44] refer to the frequency, duration, and intensity of physical activity during free time and participation in organized sports and sports competitions (e.g., Do you participate in organised physical activities, i.e., with an instructor or trainer?). Lower scores are characteristic of less active individuals, whereas higher scores are indicative of more active individuals. For its part, the intensity of physical activity performed establishes four levels: (a) Insufficient intensity; (b) Light intensity; (c) Moderate intensity; and (d) Vigorous intensity. This block also contains items that refer to whether the sports practice is carried out in federally licensed clubs or teams. The scale shows good reliability with a Cronbach’s alpha value of 0.75.

### 2.3. Procedure

Prior to administering the questionnaires, the schools sent an informative letter to the legal guardians of the minors requesting consent for participation. The letter informed about the voluntary and confidential nature of participation in the study. Subsequently, consent was confirmed with each teacher and the questionnaires were administered in the classrooms of the schools themselves, in small groups of no more than 25 students, and each participant was required to complete their questionnaire individually. The information was collected by the research staff. The students were informed of the purpose of the study and the confidential and anonymous treatment of the data. Participation in the study was voluntary, and the time required to complete the questionnaire did not exceed 15 min. Data collection took place between 26 September and 20 December 2019 during school hours. The research staff clarified any doubts the students may have had.

### 2.4. Data Analysis

Data relating to the descriptive statistics were presented as frequency (percentage) or mean (standard deviation) depending on the case. Continuous variables were analyzed by Student’s *t*-test, and categorical variables were analyzed by chi-square and proportion test. Spearman’s correlation coefficient tests were performed to test the associations between the number of cigarettes consumed per day and problematic internet use with the dependent variables “total physical activity performed” and “intensity of physical activity”. To compare the levels of physical activity and whether respondents belonged to a federated sport, we used the Fisher exact test. To obtain a measure of the effect size, Cohen’s d was used. Multiple linear regression analysis was used to examine the factors associated (physical activity, sport federated member, gender) adjusted by age with the nª of cigarettes and problematic use of the internet. The results were considered statistically significant at *p* < 0.05. The analyses were performed with STATA ver. 16.1 for Mac.

A path analysis model was developed and tested using Mplus 6 to explore the direct and indirect associations between the dependent variables. Total physical activity and Intensity of physical activity with the independent study variables, including gender and being a member of a sports federation as a mediating variable. Confirmatory factor analyses and structural models were estimated using maximum likelihood with robust corrections, according to recommended procedures for this type of data [45].

Confirmatory factor analyzes and structural models were estimated using maximum likelihood with robust corrections. To evaluate the fit of the model, the Chi-square statistic and the comparative fit index were used [46]. The measures based on the residuals (standardized root mean squared residuals) and the index of measurements in approximation errors, root mean squared error of approximation, [47,48] were also evaluated.

### 2.5. Ethical Aspects

Participants and their parents/legal guardians received information about the study and signed informed consent. All study procedures were implemented according to the Declaration of Helsinki [49] and the EDUCA Ethics Committee with approval code 42019. The anonymity of the study participants and confidentiality of the data was guaranteed.

## 3. Results

The sociodemographic and lifestyle characteristics related to tobacco use, internet use, physical activity, and intensity of physical activity are presented in Table 1. The data refer to the total sample (n = 1222), indicating gender and whether they are members of a sports federation.

Significant differences are observed according to gender for practice of physical activity (*t*(1220) = 9.029, *p* < 0.001), with an intermediate effect size (d = 0.517); as well as for level of intensity of the physical activity performed (*p* < 0.001), with an intermediate effect size (d = 0.492). In both cases, it was the boys who showed greater practice of physical activity and with higher intensities than the girls.

Statistically significant differences are also observed when considering the variable of being a member of a sports federation. The percentage of smokers is higher in the group of those who are not members of a sports federation, although with a small effect size (d = 0.119). The mean PIU score (*t*(1220) = 3.362, *p* < 0.001), with a small effect size (d = 0.204). Finally, in relation to physical activity (*t*(1220) = −26.46, *p* < 0.001) with a large effect size (d = 1.58); as well as the percentage of users who perform higher intensity physical activity (*p* < 0.001), are higher in the group of those who are members of a sports federation than those who are not.

In Spearman’s correlation analysis, we only found significance between physical activity and intensity of physical activity (ρ = 0.94, *p* < 0.001), indicating an inverse relationship with a large effect size.

Table 2 shows the relationship between the number of cigarettes consumed and its associated variables, being statistically significant for physical activity (Coef = −0.015; *p* = 0.02). The SFM and gender are not statistically significant. As can be seen in Table 2, the increase in the number of cigarettes is associated with lower levels of physical activity.

Table 3 shows the relationship between problematic internet (PIU) use and its associated variables, being statistically significant for sport federation members (Coef = −1.74; *p* = 0.001). Not being statistically significant for physical activity and gender. As can be seen in Table 3, an increase in the problematic use of the internet presents less probability of practicing federated sports. Both regressions were adjusted for age.

**Figure 1:**
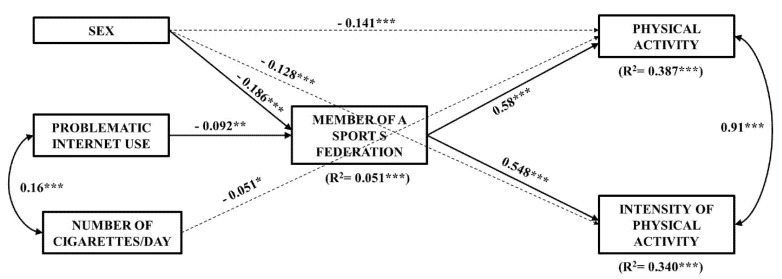
Path analysis model. Direct effects are represented by continuous arrows; indirect effects are represented by dashed arrows. The β values are shown next to each arrow. * *p* < 0.05; ** *p* < 0.01; *** *p* < 0.001.

In relation to path analysis, an initial model was proposed to predict the practice of physical activity and its intensity. The initial model was a saturated model with all possible relationships between the variables considered, showing inadequate fit indices. The final model was obtained after re-specification of the initial model, the result of which is shown in Figure 1. This respecified model obtained satisfactory fit indices: χ^2^ 104 = 486.7, *p* < 0.001; χ^2^/DF = 4.68; RMSEA = 0.055, with an interval between 0.050 and 0.060; CFI = 0.94; and SRMR = 0.04. As could be seen, all the fit indices showed scores in accordance with the criteria recommended by the specialized literature [47].

The path analysis showed that physical activity was predicted by being a member of a sports federation (β = 0.58; *p* < 0.001), sex (β = −0.141; *p* < 0.001), and by Number of cigarettes/day (β = −0.051; *p* < 0.05), explaining the variance of 38.7%. Intensity of physical activity was predicted by being a member of a sports federation (β = 0.548; *p* < 0.001) and sex (β = −0.128; *p* < 0.001), explaining the 34% variance. Likewise, being a member of a sports federation was predicted by sex (β = −0.186; *p* < 0.001) and by PIU (β = −0.051; *p* < 0.05) explaining the 5.1% variance.

In turn, the independent variables PIU and number of cigarettes consumed per day (r = 0.16; *p* < 0.001); and the dependent variables Physical activity and Intensity of physical activity (r = 0.91; *p* < 0.001) are correlated.

**Figure 2:**
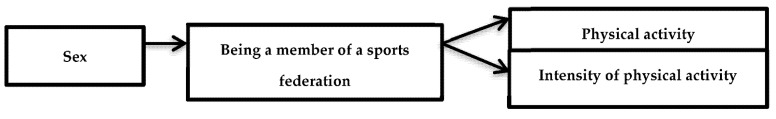
Causal model on physical activity and its intensity.

When indirect paths were analyzed, the results showed that sex predicted the criterion variable Physical activity (γ = −0.108; *p* < 0.001) through Being a member of a sports federation (Figure 2). The sex variable likewise predicted the criterion variable Intensity of physical activity (γ = −0.102; *p* < 0.001) through being a member of a sports federation (Figure 2).

**Figure 3:**
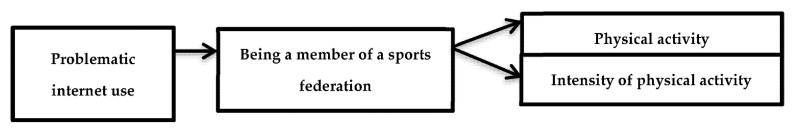
Causal model on physical activity and its intensity.

In turn, problematic internet use predicted the criterion variable physical activity (γ = −0.053; *p* < 0.01) through being a member of a sports federation (Figure 3). Similarly, problematic internet use predicted the criterion variable intensity of physical activity (γ = −0.050; *p* < 0.01) through being a member of a sports federation (Figure 3).

## 4. Discussion

The study of the relationship between PA and tobacco consumption or problematic internet use is one of the current challenges for many researchers [50,51,52]. The present study aimed to examine the relationship between unhealthy behaviors: tobacco consumption and problematic internet use in relation to PA and its intensity, analyzing the modulating role of gender and the fact of being a member of a sports federation.

Among adolescents who practice sports in a federation, there are 12.13% of smokers compared to 15.56% among those who do not practice sports in a federation, the effect size of the differences being small, with no differences found in the number of cigarettes consumed per day between the two groups. They have probably developed an addictive tobacco habit that they combine with sports practice, which is consistent with other findings (e.g., [24]). The same is true regarding problematic internet use, confirming a lower number of adolescents with internet problems in participants who play sports in a federation (14.9%) compared to those who do not (20.5%), again with a small effect size.

In relation to smoking tobacco and PIU, sex-dependent differences are found and, in this sense, a finding contrary to what is expected according to the existing literature.

With respect to sex, the ratio of boys to girls performing moderate-intensity PA was 1.7:1; and 1.6:1 for vigorous-intensity PA. Likewise, an intermediate effect size is observed in the differences observed in PA intensity, in favor of boys. These findings are in line with those that indicate higher levels of MVPA in boys than in girls [53,54,55,56], as well as the fact that during adolescence, there is a decrease in the practice of PA and sports, with a higher dropout rate in girls [57,58,59].

The data from this study show that a lower number of cigarettes consumed per day predicts greater physical activity, but it does so weakly (Figure 1), only in the case of girls (Table 3) and without being a member of a sports federation mediating this relationship (Figure 1). This finding is consistent with studies indicating a negative association [60,61]. However, the weak association observed also points in the direction of those studies that found no significant relationship [24]. In fact, it is noted that the relationship between PA and tobacco consumption may change depending on the sport modality practiced, as well as the type and intensity of PA performed. For example, participation in competitive and team sports seems to be more strongly associated with tobacco use [27,62,63,64], although some studies indicate that only in boys [63,64,65,66,67]; whereas participation in individual or endurance sports is associated with lower consumption of all substances, including tobacco [26,68].

Similarly, drug use, including tobacco, may be a response to the pressure and competitive demands associated with highly structured and rigid sports environments [67]. In this sense, it is worth highlighting the two-way nature of this relationship, considering that in addition to the health consequences that tobacco use can cause in adolescents in the medium to long term [69], it presents immediate physical consequences (e.g., reduced oxygenation capacity that affects heart and lung function) that imply a decrease in sports performance [70,71]. Thus, tobacco use favors a decrease in the practice of physical activity and sports, especially those of high intensity. This may explain the differences found between boys and girls, since tobacco would affect girls to a greater extent in their physical performance, favoring the abandonment of practicing PA.

Regarding the fact that PIU negatively predicts PA and its intensity, the results of the present study indicate that there is a weak relationship between the practice of physical activity and its intensity, being observed only in girls, but in the opposite direction to that expected (Table 3). This finding is novel and contrary to that reported by Costigan et al. [72] who indicated a small negative association of sedentary screen time with the practice of PA. In our study, it is found that, in the case of girls, a higher PIU predicts a higher practice of PA and PA intensity, although with a small effect size. This finding can be explained by the Compensatory carry-over action model [73], which is based on the belief that certain unhealthy (but pleasurable) behaviors can be compensated by engaging in other healthier behaviors [74].

The first variable, sex, negatively predicts PA and its intensity, both directly and indirectly through being a member of a sports federation or not (Figure 1 and Figure 2). That is, boys practice more PA and at a higher intensity, regardless of whether or not they participate in a sports federation. This finding may be explained by a variety of social and educational factors, such as gender stereotypes of a corporal or competitive nature. Beliefs establish that boy attractiveness lies in a strong and vigorous body and that boys physical abilities are superior to those of girls. These stereotypes are observed more in boys than in adolescent girls [69], which can be a motivator for the practice of PA. On the other hand, for boy athletes, certain behaviors and actions are linked to status and position in the social hierarchy, including tobacco use [22].

The second variable, PIU, negatively predicts PA and its intensity only indirectly through being a member of a sports federation or not (Figure 1 and Figure 3). That is, it is the adolescents who do not show PIU who practice more PA and with greater intensity, but only if it is through being a member of a sports federation. This fact highlights the role of promoting PA through structured environments that require consistency and commitment as a strategy to prevent sedentary lifestyles associated with screens.

This study also contributes to the positive relationship observed between PIU and tobacco use as a complementary finding (Figure 1), in line with numerous investigations [70,71]. In this regard, recent research has observed that social anxiety is a predictor of tobacco use [75] and PIU [76]. It’s a line of interest to analyze the role of this variable in the sports field, where competitive pressure and demand could trigger social anxiety and, consequently, explain to some extent the consumption of tobacco and PIU in athletes.

Based on the findings of this study, we consider that the practice of PA should be considered from the socio-ecological model [77], contributing to a holistic view of the interaction of intrapersonal, interpersonal, institutional, community, and public policy factors. The practice of PA should be integrated into a multibehavioral health promotion model, rather than addressing these behaviors in isolation [78]. Several systematic reviews and studies [79,80] point in this direction, having demonstrated greater efficacy and impact at the preventive level. According to the postulates of the TAD self-determination theory [81], the support of different social agents (e.g., parents, PE teachers, sports club coaches, etc.) can influence the motivational processes of adolescents toward the practice of PA [82,83]. These same agents, duly trained, can provide brief advice on other health issues such as drug use (legal and illegal) and internet use.

### Limitations of the Study and Recommendations for Future Research

This study had different limitations that may affect the generalization of the results. The origin of the sample makes the findings conditioned to cultural factors that can determine lifestyles. As it was a cross-sectional design, it was not possible to establish causal relationships between the variables, concluding in terms of an association between them, although their bidirectionality was based on the existing literature. The results are based on self-reports by the participants, and response bias (e.g., dissimulation) may exist. Although these self-reports are considered valid and reliable instruments for data collection, it would be advisable to use other instruments in order to compare the results obtained. Lastly, the development of longitudinal studies throughout adolescence is desirable in order to establish causality between the variables under study, as well as their possible variation in the temporal spectrum of the adolescent stage.

## 5. Conclusions

The results of this study confirm a significant positive relationship between physical activity, intensity, and the practice of sports with a federation, as well as between cigarette consumption and internet use. Based on these findings, it is proposed to implement public policies that promote the practice of sports as a direct investment in health by preventing tobacco consumption and other harmful habits for adolescents, such as sedentary screen time, or more specifically, PIU.

Furthermore, although it is assumed that the practice of sports in a federation is a supervised health-promoting environment, it has been found that there are adolescent tobacco users in this setting. In this sense, a review of sports federation models is considered necessary, analyzing whether certain aspects associated with the culture surrounding sports practice are encouraging substance use behaviors among young people. Furthermore, the need to include educational and supervisory aspects of health issues in sports settings with adolescents.

Finally, it is essential to consider the gender perspective in a transversal manner in policies and actions aimed at promoting health. In this sense, equity would reduce the vulnerability of girls to various health risks during adolescence and into adulthood.

## Figures and Tables

**Table 1:** Descriptive characteristics of the participants.

	Total n (%)	Boys n (%)	Girls n (%)				SPFE_NOT n (%)	SPFE_YES n (%)			
	1222 (100)	579 (47.4)	643 (52.6)	*z* @	*p*	*d*	752 (61.5)	470 (38.5)	*z* @	*p*	*d*
Tobacco user, n (%)	174 (14.24)	76 (13.13)	98 (15.24)	−1.05	0.29		117 (15.56)	57 (12.13)	1.66	0.09	0.119
Cigarettes per day				*χ*^2^ #	*p*				*χ*^2^ #	*p*	
None, n (%)	1048 (85.8)	503 (86.9)	545 (84.8)	2.4	0.3		635 (84.4)	413 (87.9)	2.79	0.247	
Less than 10 cigarettes/day, n (%)	144 (11.8)	60 (10.4)	84 (13.1)				97 (12.9)	47 (10.0)			
11 or more cigarettes/day, n (%)	30 (2.4)	16 (2.7)	14 (2.1)				20 (2.7)	10 (2.1)			
				*t* $	*p*	*d*			*t* $	*p*	*d*
Problematic internet use, m (SD)	9.85 (6.92)	9.55 (6.90)	10.11 (6.93)	−1.399	0.162	−0.08	10.37 (7.19)	9.01 (6.38)	3.36	<0.001	0.204
Total physical activity, m (SD)	9.75 (2.31)	10.35 (2.23)	9.20 (2.24)	9.03	<0.001	0.517	8.64 (1.9)	11.51 (1.74)	−26.46	<0.001	1.59
PA Intensity				*χ* ^2^ *#*	*p*	*d*				*p* &	
Insufficient PA intensity, n (%)	251 (20.5)	71 (12.3)	180 (28.0)	69.81	<0.001	0.492	244 (32.4)	7 (1.5)		<0.001	
Light PA intensity, n (%)	489 (40.0)	218 (37.6)	271 (42.1)				370 (49.2)	119 (25.3)			
Moderate PA intensity, n (%)	425 (34.8)	256 (44.2)	169 (26.3)				134 (17.8)	291 (61.9)			
Vigorous PA intensity, n (%)	57 (4.7)	34 (5.9)	23 (3.6)				4 (0.5)	53 (11.3)			

Note: SPFE_NOT: sport federation non member; SPFE_YES: sport federation member; #: chi-squared test; $: *t*-test; @: Pr-test (proportion test); &: Fisher’s exact test.

**Table 2:** Linear regression between the number of cigarettes consumed and its associated variables. Adjusted by age.

Variables	Coef	95% CI	Std. Err.	*t*	*p*
PA	−0.015	(−0.02—−0.002)	0.006	−2.29	0.02
Sport federation member	0.017	(−0.04—0.07)	0.031	0.55	0.58
Gender	−0.006	(−0.05—0.4)	0.025	−0.28	0.78

**Table 3:** Linear regression between problematic internet use and its associated variables. Adjusted by age.

Variables	Coef	95% CI	Std. Err.	*t*	*p*
PA	0.18	(−0.02—0.39)	0.10	1.72	0.08
Sport federation member	−1.74	(−2.7—−0.74)	0.50	−3.43	0.001
Gender	0.40	(−0.39—1.20)	0.40	0.99	0.32

## Data Availability

Data sharing not applicable.
